# Phase 1 trial of olaparib and oral cyclophosphamide in *BRCA* breast cancer, recurrent *BRCA* ovarian cancer, non-*BRCA* triple-negative breast cancer, and non-BRCA ovarian cancer

**DOI:** 10.1038/s41416-018-0349-6

**Published:** 2019-01-17

**Authors:** Chee Khoon Lee, Clare Scott, Geoffrey J. Lindeman, Anne Hamilton, Elizabeth Lieschke, Emma Gibbs, Rebecca Asher, Heath Badger, Robin Paterson, Lauren Macnab, Edmond Michael Kwan, Prudence A. Francis, Frances Boyle, Michael Friedlander

**Affiliations:** 1 St George Hospital, Sydney, NSW Australia; 2 National Health and Medical Research Council (NHMRC) Clinical Trials Centre, Sydney, NSW Australia; 3 Peter MacCallum Cancer Centre, Melbourne, VIC Australia; 4 Walter and Eliza Hall Institute of Medical Research, Melbourne, VIC Australia; 5 The Royal Melbourne Hospital, Melbourne, VIC Australia; 6 Breast Cancer Trials Australia & New Zealand, Newcastle, NSW Australia; 7 The Mater Hospital, North Sydney, NSW Australia; 8 Prince of Wales Hospital, Sydney and Prince of Wales Clinical School, University of New South Wales, Sydney, NSW Australia

**Keywords:** Cancer, Oncology

## Abstract

**Background:**

We conducted a Phase 1 study to evaluate safety and activity of olaparib tablets and oral cyclophosphamide.

**Methods:**

Patients had metastatic breast cancer (BC) or recurrent high-grade serous ovarian cancer (HGSOC), performance status 0–2, and ≤3 lines of prior therapy. Patients were treated using a dose escalation strategy with cohort expansion once maximal tolerated dose (MTD) was determined. Dose level 1 (DL1): olaparib 300 mg bid, cyclophosphamide 50 mg on days 1, 3, and 5, weekly. DL2: olaparib 300 mg bid, cyclophosphamide 50 mg, days 1–5 weekly.

**Results:**

Of 32 patients, 23 had HGSOC (germline *BRCA* mutation [g*BRCA*m] 70%) and 9 had BC (g*BRCA*m 67%). Four were treated at DL1 and 28 at DL2, the MTD. Haematological adverse events (AEs) were most common: grade 3/4 AEs: lymphopenia 75%, anaemia 31%, neutropenia 37%, thrombocytopenia 47%. Two permanently discontinued treatment due to haematological AEs. In BC, no objective response was reported. Unconfirmed objective response was 48% and 64% for all HGSOC and g*BRCA*m subset, respectively. CA125 responses were 70% (all HGSOC) and 92% (g*BRCA*m).

**Conclusions:**

In HGSOC and BC, olaparib 300 mg bid and cyclophosphamide 50 mg on days 1–5 weekly were tolerable and active, particularly in g*BRCA*m, and is worthy of further investigation.

## Introduction

Olaparib is a first-in-class potent oral poly (ADP-ribose) polymerase (PARP) inhibitor. Monotherapy studies have demonstrated acceptable toxicity and activity in metastatic breast cancer^1–5^, and recurrent ovarian cancer^1–4,6,7^ in patients with germline *BRCA* mutations (g*BRCA*m). Low-grade nausea, vomiting, fatigue, and anaemia were the most frequently reported adverse events (AEs). Response rates varied from 0 to 60% and 26 to 41% for g*BRCA*m breast and ovarian cancer patient cohorts, respectively.

Inhibition of base-excision DNA repair by olaparib potentiates the DNA damage and cytotoxicity caused by platinum chemotherapy, leading to an increase in genomic instability and tumour cell death.^8,9^ Indeed, studies of the combination of olaparib and platinum chemotherapy followed by maintenance olaparib have reported high response rates and prolonged progression-free survival (PFS)^10^ for g*BRCA*m recurrent ovarian carcinomas but with a significant increase in myelotoxicity^10–12^ requiring a reduction of the doses of both carboplatin and olaparib for safe administration, such that the combination is not recommended. Similar findings were reported in metastatic triple-negative breast cancer, where combination of olaparib with paclitaxel resulted in high rates of neutropenia despite secondary prophylaxis with granulocyte-colony stimulating factor.^13^

Cyclophosphamide is a bifunctional alkylating agent with modest monotherapy activity in advanced breast^14,15^ and ovarian cancers.^16^ Cyclophosphamide damages DNA through the formation of complex inter-strand crosslinks, which could potentiate the activity of PARP inhibitors and lead to greater cytoxicity.^17^ Pre-clinical studies also provide some evidence to support combination therapy with alkylating chemotherapy and PARP inhibitors, particularly those that trap PARP–DNA complexes effectively.^18,19^ Metronomic cyclophosphamide is administered orally at a low dose on a continuous schedule for prolonged periods and is well tolerated with low rates of myelosuppression. Therefore, theoretically, metronomic cyclophosphamide could be combined with a PARP inhibitor without associated significant toxicity and with increased cytotoxicity.

However, a randomised Phase 2 trial of combination veliparib and metronomic oral cyclophosphamide in heavily pre-treated ovarian cancer patients failed to demonstrate improvement in either the response rate or PFS over cyclophosphamide monotherapy.^20^ Veliparib is a less efficient trapper of PARP than olaparib,^18^ however, and this trial used a potentially subtherapeutic dose of veliparib 60 mg daily, compared to the recommended monotherapy dose of 300–400 mg twice daily, in order to enable continuous cyclophosphamide 50 mg daily dosing.

We conducted the SOLACE trial, a Phase 1 study of olaparib tablets and metronomic oral cyclophosphamide in pre-treated patients with metastatic g*BRCA*m breast cancer, recurrent g*BRCA*m high-grade serous ovarian cancer (HGSOC), non-*BRCA* triple-negative breast cancer, and non-*BRCA* HGSOC. The objectives of the SOLACE trial were to establish the safety, tolerability, maximum tolerated dose (MTD), and preliminary efficacy of the combination. Unlike the previous veliparib-cyclophosphamide study, our trial adopted the strategy of maintaining the recommended monotherapy dose of olaparib tablets while escalating the metronomic administration of oral cyclophosphamide, using a dose-escalation design.

## Patients and methods

The SOLACE trial (ANZCTRN: 12613000924752) was an investigator-initiated study sponsored by Breast Cancer Trials Australia and New Zealand. Astrazeneca provided olaparib for this study. Ethical approval for the conduct of the study was provided by the appropriate Human Research Ethics Committees at each of the three participating clinical sites. All patients provided written informed consent.

### Eligibility criteria

Eligible patients with recurrent or metastatic disease after standard therapies had measurable (Response Evaluation Criteria in Solid Tumors [RECIST] v1.1) or non-measurable HGSOC with an elevated CA125 evaluable using Gynecologic Cancer InterGroup (GCIG) CA125 response criteria,^21^ an Eastern Cooperative Organization Group performance status of ≤2, and adequate bone marrow, liver, and kidney function. Documentation of g*BRCA*m status was not required. Prior exposure to a PARP inhibitor was not allowed. Patients who were unable to swallow pills or those with uncontrolled intercurrent illness, including brain metastases or gastrointestinal conditions that might predispose to drug intolerance or poor drug absorption were also excluded.

### Study design

The trial design used a backbone of olaparib tablets 300 mg twice daily continuously for all patients starting on day 1 of a 3-weekly cycle. Oral cyclophosphamide 50 mg was given, starting on day 1 of a 3-weekly cycle, on an increasing number of days each week (dose level (DL) 1 for 3 days, DL2 for 5 days, and DL3 for 7 days) to sequential cohorts of patients (Table 1). Dose levels with lower olaparib doses were included in the design in the event of unexpected toxicity, but these were not activated. Eligible patients were enrolled in a ‘3 + 3’ dose escalation format, with cohort expansion at the MTD.

**Table 1:** Patient cohort at different dose levels

DL	Olaparib tablet	Cyclophosphamide tablet	Number of patients
−2	200 mg bid	50 mg, days 1, 3 and 5 every week	0
−1	250 mg bid	50 mg, days 1, 3 and 5 every week	0
1	300 mg bid	50 mg, days 1, 3 and 5 every week	4
2	300 mg bid	50 mg, days 1–5 every week	28^a^
3	300 mg bid	50 mg, days 1–7 every week	0

*DL* dose level, *mg* milligram, *bid* twice per day

^a^7 patients were initially enrolled in DL2. When this was determined to be the MTD, another 21 patients were enrolled in DL2 as a separate expansion cohort

Patients were evaluated for toxicity and response over 24 weeks or 8 cycles of treatment, with each treatment cycle administered over 3 weeks. Toxicity was graded using the Common Terminology Criteria for Adverse Events v4.0, and tumour response was assessed at weeks 6, 15, and 24; response and progression was evaluated using RECIST v1.1 for patients with measurable disease and CA125 (for the HGSOC cohort) as defined by GCIG criteria.^21^ Study treatment was discontinued for symptomatic disease progression, intercurrent illness, unacceptable toxicity, or patient withdrawal of consent. In the absence of disease progression, patients were allowed to continue with one or both study agents beyond 24 weeks at the discretion of the study physicians. Patients were also allowed to continue study treatment in the presence of radiological or CA125 progression for asymptomatic or minimally symptomatic patients at the discretion of the study physicians.

### Definitions of dose-limiting toxicity and maximum tolerated dose

The primary endpoint of this study was to determine the recommended Phase 2 dose (RP2D) of the olaparib and cyclophosphamide combination, defined by the MTD or the highest protocol-defined dose in the absence of dose-limiting toxicity (DLT). DLT was any of the following events that occurred during the first 6 weeks of therapy: neutrophil count < 0.5 × 10^9^/L without fever and lasting for >5 days, neutropenic sepsis, platelet counts < 25 × 10^9^/L, any grade 3 or 4 non-haematological adverse event (AE) despite appropriate supportive measures, any AE not otherwise described that resulted in a treatment delay of >21 consecutive days and repeated requirement for blood transfusions within the first 2 cycles (6 weeks). In the determination of the MTD, recurrent AEs that were encountered beyond the first 6 weeks of therapy were also taken into consideration (Supplementary Table 1).

### Pharmacokinetic studies

For the DL1 cohort, plasma samples to quantify serum olaparib level were collected 1, 2, 6, and 12 h after ingestion of the morning dose of olaparib on day 7 (following 1 week of olaparib monotherapy). Plasma samples were collected at the same time points on day 14 (after patients had received 1 week of combination therapy with olaparib and cyclophosphamide). For the DL2 cohort, samples were collected at the same time points on days 8 of cycles 1 and 2 for patients receiving combination olaparib–cyclophosphamide. Blood samples were centrifuged and stored until measurement using validated assays with a lower limit of quantitation of 0.02 µg/mL for olaparib.^22^

### Statistical analyses

Baseline demographics were summarised using the median number and range for continuous variables, and frequency and percentages for categorical variables. All patients treated with at least one dose of the study drugs were evaluated for efficacy and toxicity. For efficacy analyses, objective response rates (ORR) and stable disease for at least 6 weeks (SD6) were computed separately for breast and HGSOC cohorts as well as for the g*BRCA*m cohorts regardless of tumour types. ORR was defined as the sum of complete responses and partial responses. DCR was defined as the sum of complete responses, partial responses and stable disease for ≥6 weeks. Response duration was not evaluated due to infrequent nature of tumour assessments (weeks 6, 15, and 24 only). The number of patients with each type of worst grade toxicity experienced was summarised as percentages. PFS duration was defined as the time from study commencement to objective tumour progression or death. The Kaplan–Meier approach was used for analysis of PFS.

## Results

### Patient characteristics

Between June 2014 and August 2016, 32 women were enrolled (dose escalation cohort: *N* = 11; dose expansion cohort: *N* = 21), with 16 patients still receiving olaparib monotherapy at the time of data cut-off in September 2017. Table 2 summarises the baseline patient characteristics. HGSOC was the most common tumour type (*N* = 23 [72%]), and the majority of these patients had g*BRCA*m (overall *N* = 22 [69%], HGSOC *N* = 16, breast cancer *N* = 6). Patients had a good performance status, with a median age of 56 years. A total of 28% of patients had received three lines of systemic therapy; Supplementary Table 2 summarises in detail the different types of prior therapy.

**Table 2:** Baseline characteristics of the trial participants

	Characteristics	Breast cancer *N* (%)	HGSOC *N* (%)
	Sex		
	Female	9 (100)	23 (100)
	Age (years)		
	Median	46	60
	Range	33–64	37–84
	ECOG performance status		
	0	6 (67)	13 (57)
	1	3 (33)	10 (43)
	*gBRCA* status		
	* BRCA* negative	3 (33)	6 (26)
	* gBRCA* 1	2 (22)	10 (44)
	* gBRCA* 2	4 (45)	6 (26)
	Unknown	0	1 (4)
	Breast Cancer cohort		
	ER positive	6	
	PR positive	4	
	ER, PR, and HER2 negative	3	
	Lines of prior therapy		
	1	2 (22)	9 (39)
	2	4 (45)	8 (35)
	3	3 (33)	6 (26)
	Breast Cancer cohort		
	Prior anthracycline	7 (78)	
	Prior taxane/anti-tubulin	8 (89)	
	Prior platinum^a^	6 (67)	
	Ovarian Cancer cohort		
	Lines of prior platinum-based therapy (1/2/3)		9 (39)/10 (44)/4 (17)
	Prior bevacizumab		6 (26)
	Platinum-sensitive at first relapse		22 (96)
	Platinum-sensitive at trial enrolment		15 (65)

*ECOG* Eastern Cooperative Organization Group, *HGSOC* high-grade serous ovarian cancer

^a^Only 1 patient with germline *BRCA* mutation did not receive prior platinum chemotherapy

### Dose optimisation and treatment-related adverse events

We have identified the RP2D as olaparib tablets 300 mg twice daily, and cyclophosphamide tablets 50 mg on days 1–5 each week. Despite no DLT’s being observed with olaparib plus cyclophosphamide during the first 6 weeks of therapy at DLs 1 and 2, patients did not proceed to DL3 due to recurrent haematological AEs observed beyond 6 weeks (Table 1). A total of 16 (50%) patients stopped treatment before or at 24 weeks; only two patients stopped early due to unacceptable toxicity. The remaining 16 patients (50%) continued with study treatment beyond 24 weeks; all of these patients continued olaparib monotherapy after stopping cyclophosphamide. The median duration of protocol-defined therapy during the eight cycles was 5.5 months (range: 0.7–5.5). Among patients who continue olaparib beyond the eighth cycle, the median duration of therapy was 5.0 months (range: 0.7–23.5 + ).

**Fig. 1:**
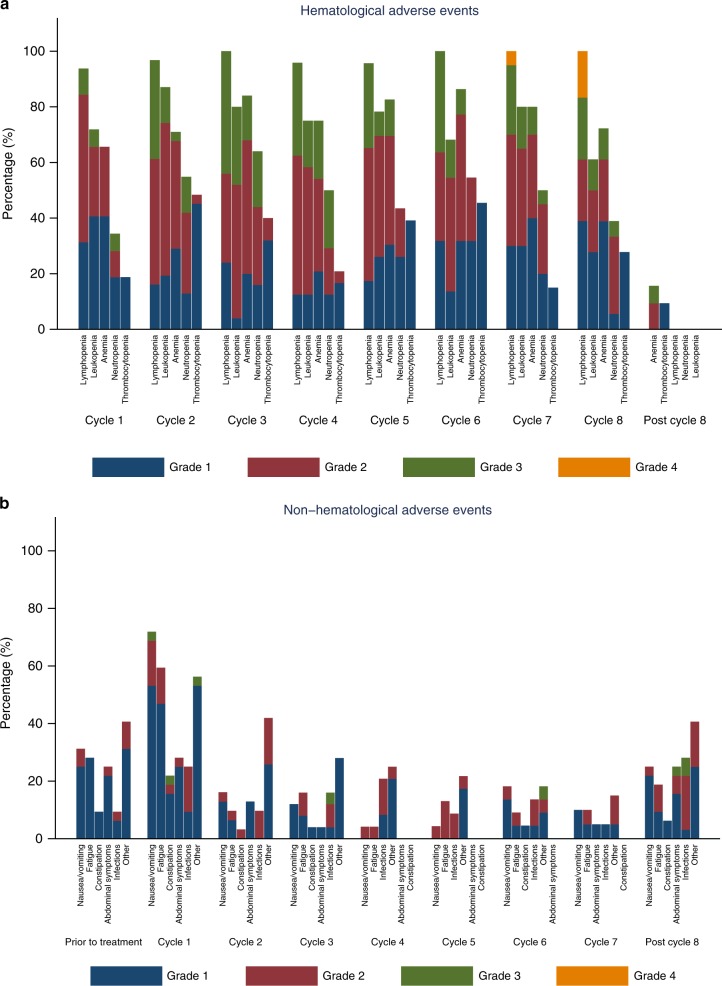
Frequency of adverse events according to **a** haematological and **b** non-haematological types experienced after each treatment cycle

Table 3 summarises any-grade AE’s in all patients. Grades 3 and 4 AE’s occurred in 84 and 13% of patients, respectively. Haematological AE’s were the most common: lymphopenia (grade 1/2: 25%, grade 3/4: 75%), anaemia (grade 1/2: 57%, grade 3: 31%), neutropenia (grade 1/2: 44%, grade 3/4: 37%), and thrombocytopenia (grade 1/2: 47%, grade 3/4:47%). A total of 50% of patients required a blood transfusion for anaemia during the course of the study. No febrile neutropenia was reported. The most common non-haematologic AE’s were nausea/vomiting (grade 1/2: 91%, grade 3: 3%), fatigue (grades 1/2: 84%), and constipation (grade 1/2: 34%, grade 3: 3%). Grades 3 and 4 AE’s did not differ significantly between the breast and HGSOC cancer cohorts (*P* = 0.99). Supplementary Table 3 summarises worst grade AE’s for different DL. Fig. 1 summarises the (A) haematological and (B) non-haematological AE’s experienced after each treatment cycle. The frequency of haematological AE’s increased with each treatment cycle, while the frequency of non-haematological AE’s decreased with each cycle. Supplementary Tables 4 and 5 provide the details of dose modifications for olaparib and oral cyclophosphamide due to haematological AEs.

**Table 3:** Treatment-related adverse events by maximum grade per patient

	Grade 1	Grade 2	Grade 3	Grade 4	Total
Lymphocyte count decrease	2 (6%)	6 (19%)	21 (66%)	3 (9%)	32
Nausea/vomiting	21 (66%)	8 (25%)	1 (3%)		30
White blood count decrease	3 (9%)	12 (38%)	14 (44%)	1 (3%)	30
Anaemia	4 (13%)	14 (44%)	10 (31%)		28
Fatigue	17 (53%)	10 (31%)			27
Neutrophil count decrease	2 (6%)	12 (38%)	11 (34%)	1 (3%)	26
Infection	3 (9%)	15 (47%)	3 (9%)		21
Platelet count decrease	16 (50%)	2 (6%)			18
Abdominal pain, bloating, dyspepsia, distension	12 (38%)	2 (6%)	1 (3%)		15
Non-abdominal pain	13 (41%)	2 (6%)			15
Constipation	9 (28%)	2 (6%)	1 (3%)		12
Headache	10 (31%)	1 (3%)			11
Dizziness	10 (31%)				10
Dysgeusia	10 (31%)				10
Cough	7 (22%)	2 (6%)			9
Diarrhoea	8 (25%)		1 (3%)		9
Anorexia	7 (22%)	1 (3%)			8
Dyspnoea	7 (22%)	1 (3%)			8
Oral mucositis	8 (25%)				8
Fever	3 (9%)				3
Hypertension	1 (3%)	1 (3%)	1 (3%)		3
Decrease in serum phosphate		3 (9%)			3
Nail changes	2 (6%)				2
Vascular disorders	1 (3%)	1 (3%)			2
Decrease in serum potassium	1 (3%)				1
Decrease in serum magnesium		1 (3%)			1
Decrease in serum sodium	1 (3%)				1
Increase in serum creatinine			1 (3%)		1

Two (6%) patients, treated at DL2, permanently discontinued treatment due to toxicity for anaemia (during cycle 5), and thrombocytopenia (during cycle 6), respectively. A total of 18 patients (56%) completed eight cycles of protocol-defined therapy; 16 of these patients (50%) continued beyond eight cycles outside protocol at the discretion of the study investigators. A total of 12 patients (38%) terminated treatment before eight cycles of protocol-defined therapy due to PD.

### Preliminary efficacy

**Fig. 2:**
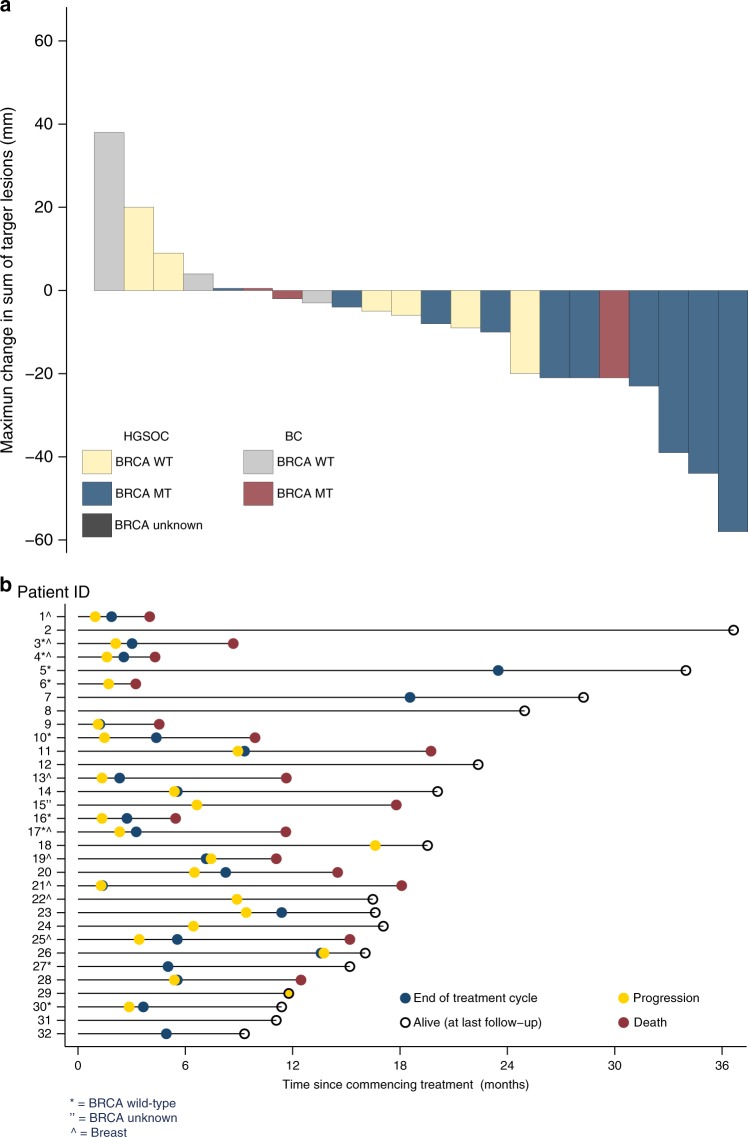
**a** Best response for target lesions by patient based on maximal percentage of tumour reduction. **b** Swimmer plot depicts individual patients as lines. BC metastatic triple-negative breast cancer, HGSOC high-grade serous ovarian cancer

**Fig. 3:**
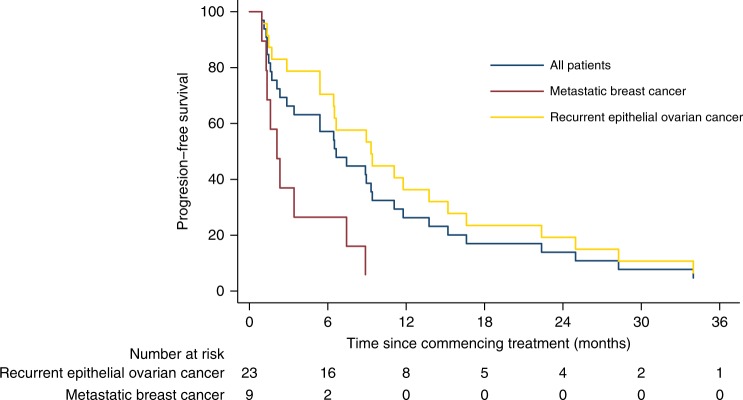
Kaplan–Meier curves for progression-free survival for the overall population and the breast and ovarian cancer cohorts

In the ovarian cancer cohort (*N* = 23; g*BRCA*m *N* = 16), 21 patients (g*BRCA*m *N* = 14) had evaluable disease by RECIST and 20 patients (g*BRCA*m *N* = 13) were evaluable by GCIG CA125 criteria. Changes from baseline in tumour size and duration of response are shown in Fig. 2. ORR was 48% (10/21) overall, and 64% (9/14) in those with g*BRCA*m. SD6 was 81% (17/21) overall, 93% (13/14) in those with g*BRCA*m. GCIG CA125 response rate was 70% (14/20) overall, and 92% (12/13) in those with g*BRCA*m. Median PFS was 9.4 months overall, and 11.8 months in the g*BRCA*m subset (Fig. 3). Supplementary Table 6 shows the relationship with response and platinum sensitivity in the ovarian cancer cohort.

In the breast cancer cohort (*N* = 9, g*BRCA*m *N* = 6), all patients were evaluable by RECIST. No objective responses were observed, but SD6 was 56% (5/9) overall, and 50% (3/6) in those with g*BRCA*m. Median PFS was 2.1 months overall, and 1.3 months in the g*BRCA*m subset (Fig. 3).

### Pharmacokinetic profiles

Pharmacokinetic (PK) data were available for five patients from the DL1 cohort. Before cyclophosphamide co-administration, the area under olaparib concentration curve (AUC) over time for olaparib monotherapy was 52.5 (95% CI: 44.5–61.9). There were no significant differences in the AUC with olaparib and cyclophosphamide co-administration (49.5, 95% CI: 42.1–58.1) (Supplementary Figure 1A). In the larger extension cohorts (*N* = 19), the AUCs with olaparib and cyclophosphamide co-administration were 55.8 (95% CI: 40.5–76.7) at day 7 of cycle 1 and 53.3 (95% CI: 39.2–72.5) at day 7 of cycle 2, respectively (Supplementary Figure 1B).

## Discussion

In this study, we have demonstrated that co-administration of olaparib tablets at the recommended dose of 300 mg twice daily with metronomic oral cyclophosphamide 50 mg on days 1–5 weekly is safe and tolerable in patients with metastatic breast cancer and recurrent HGSOC. Our study utilised olaparib tablets rather than the capsule formulation (sixteen capsules daily) used in earlier trials; the dose of 300 mg twice daily in tablet form (four tablets daily) has been established to be equivalent to 400 mg twice daily in capsule form^23^ and is much more convenient for patients. Although no protocol-defined DLT events occurred during the first two cycles, no patient was recruited into the DL3 cohort because of concerns of haematological toxicity observed in patients in the later cycles. Despite the small number of patients and limited data availability, our pharmacokinetic studies suggested that olaparib exposure did not decrease with co-administration of metronomic oral cyclophosphamide. This study established the recommended RP2D dose of olaparib tablets as being 300 mg twice daily, and cyclophosphamide tablets 50 mg on days 1–5 weekly.

Myelosuppression was the most significant toxicity associated with combination olaparib–cyclophosphamide therapy. In our study, we observed high rates of grade 3 anaemia (31%), grade 3/4 neutropenia (37%), grade 3/4 lymphopenia (75%) and grade 3/4 thrombocytopenia (47%). Although 50% of patients required a blood transfusion for anaemia during the course of the study, there was no reported febrile neutropenia. In contrast, previous olaparib monotherapy studies^1–7^ reported rates of grade 3/4 anaemia ranging from 3 to 17%, neutropenia of 9%, lymphopenia of 8 to 12% and no grade 3/4 for thrombocytopenia. Oral cyclophosphamide monotherapy is associated with low rates of myelosuppression with one recent study reporting 8% of grade 3 lymphopenia as the most significant haematological toxicity.^20^ In a randomised Phase 2 trial, veliparib (60 mg daily) and oral cyclophosphamide (50 mg daily) was associated with rates of grade 3 anaemia of 5%, neutropenia of 5%, lymphopenia of 35%, and thrombocytopenia of 5%.^20^ Importantly, that study investigated veliparib at a dose that is significantly lower than used in current trials. For example, veliparib doses have ranged from 250 to 400 mg twice daily when used either as monotherapy or in combination with platinum-based chemotherapy. It is therefore unclear whether the dose of veliparib 60 mg daily has any therapeutic impact on any g*BRCA*m and other homologous recombination-deficient cancers.

Non-haematologic toxicities, namely nausea/vomiting, fatigue, and constipation were generally of low grades. Although these low-grade toxicities might have a major impact in patients undergoing continuous treatment administered chronically, our trial suggests that the frequency and severity of these toxicities decrease over time (Fig. 1b). Importantly, the two patients (6%) who permanently discontinued the study treatment due to AEs were haematological rather than non-haematological in nature.

This study has also demonstrated promising preliminary evidence of anti-tumour activity with a combination of olaparib and metronomic cyclophosphamide in the HGSOC cohort. No significant activity was demonstrated in the breast cancer cohort, but the small number of patients treated precludes any conclusion regarding efficacy in this group.

This study has several strengths. We adopted the strategy of maintaining the recommended monotherapy dose of olaparib tablets while modifying the scheduling of cyclophosphamide. In keeping with data from multiple studies, the efficacy of olaparib monotherapy in homologous recombination-deficient cancers has already been well demonstrated, and hence our treatment strategy avoided any compromise in olaparib dosing. Unlike previous studies that utilised platinum and other chemotherapeutic agents, which were limited due to significant myelotoxicity, our choice of oral cyclophosphamide as the DNA-damaging agent has revealed acceptable tolerability despite higher rates of anaemia, neutropenia, and lymphopenia than reported with olaparib monotherapy. However, there are also several limitations. It is not possible to draw definitive conclusions regarding efficacy of olaparib and metronomic oral cyclophosphamide combination in this phase 1 trial. We do not fully understand whether the apparent synergistic anti-tumour activity with this combination is mediated through enhanced PARP trapping with olaparib.

In conclusion, olaparib and metronomic oral cyclophosphamide is relatively well-tolerated, with an acceptable safety profile. Owing to the encouraging preliminary evidence of efficacy, a multi-centre, randomised Phase 2 study in patients with advanced HGSOC with and without g*BRCA*m will commence shortly.

## Electronic supplementary material

Pharmacokinetics

Appendix Table 1 - Dose-limiting toxicity definition

Appendix Table 2 - Prior systemic therapy

Appendix Tble 3 - Worst grade adverse events overall and by patient cohort at different dose levels

Appendix Table 4 - Reduction in olaparib dose in different treatment cycle due to Grade 3 or 4 anaemia, neutropenia or thrombocytopenia

Appendix Table 5 - Reduction in oral cyclophosphamide dose in different treatment cycles due to Grade 3 or 4 anaemia, neutropenia or thrombocytopenia are described below. In each case, the cyclophospha

Appendix Table 6 - Relationship with response and platinum sensitivity in the ovarian cancer cohort only
