# Supplementary material for: Phase 1 trial of olaparib and oral cyclophosphamide in BRCA breast cancer, recurrent BRCA ovarian cancer, non-BRCA triple-negative breast cancer, and non-BRCA ovarian cancer
Source: Br J Cancer. 2019 Jan 17;120(3):279–85. doi: 10.1038/s41416-018-0349-6 (PMC6353881; doi:10.1038/s41416-018-0349-6)
Supplement: Supplementary file 2 — Appendix Table 1 - Dose-limiting toxicity definition [file 41416_2018_349_MOESM2_ESM.docx]

**Appendix Table 1:** Dose-limiting toxicity definition

In our protocol, we have defined DLT as any of the following events that occur during the first two cycles:

- Actual neutrophil count (ANC) <0.5 x 10^9^/L without fever and lasting for more than five days
- ANC >0.5 × 10^9^/L but less than 1.5 × 10^9^/L with fever ≥38.5^o^C and neutropenic sepsis
- Platelets <25 x 10^9^/L
- Any Common Terminology Criteria for Adverse Events (CTCAE) version 4.0 Grade 3 or 4 non-hematological AEs with the exception of fatigue, nausea, vomiting, diarrhea, myalgia or arthralgia, unless appropriate measures have been undertaken to treat these symptoms
- Any AEs not otherwise described that results in treatment delay of >21 consecutive days
- Any Grade 3 or 4 AEs considered, in the opinion of the investigator, to be dose-limiting
- Requirement for repeated blood transfusions within the first two cycles.

The occurrence of any of the above DLT beyond Cycle 2 for the current or previous dose levels will also be considered by the Trial Management Committee at the time of any decision to escalate or expand recruitment for a dose level.

The determination of the MTD is based on the last dose level before occurrence of the DLT.
