# Supplementary material for: Phase 1 trial of olaparib and oral cyclophosphamide in BRCA breast cancer, recurrent BRCA ovarian cancer, non-BRCA triple-negative breast cancer, and non-BRCA ovarian cancer
Source: Br J Cancer. 2019 Jan 17;120(3):279–85. doi: 10.1038/s41416-018-0349-6 (PMC6353881; doi:10.1038/s41416-018-0349-6)
Supplement: Supplementary file 3 — Appendix Table 2 - Prior systemic therapy [file 41416_2018_349_MOESM3_ESM.docx]

**Appendix Table 2:** Prior systemic therapy

|  | |  | Epithelial Ovarian Cancer  N (%) | Metastatic Breast Cancer  N (%) |
| --- | --- | --- | --- | --- |
| Adjuvant therapy | | | | |
| AC and paclitaxel | | |  | 1 (11.1) |
| AC-T | | |  | 2 (22.2) |
| Carboplatin and gemcitabine | | | 1 (4.4) |  |
| Cisplatin | | |  | 1 (11.1) |
| Docetaxol and cyclophosphamide | | |  | 1 (11.1) |
| FEC-D-Capecitabine | | |  | 1 (11.1) |
| FEC-T | | |  | 1 (11.1) |
| FEC | | |  | 1 (11.1) |
| Paclitaxel and carboplatin | | | 19 (82.6) | 1 (11.1) |
| Paclitaxel, carboplatin and bevacizumab | | | 3 (13.0) |  |
| First-line therapy post-disease relapse | | | | |
|  | Capecitabine | |  | 1 (14.3) |
|  | Carboplatin | | 1 (7.1) | 1 (14.3) |
|  | Carboplatin and gemcitabine | |  | 1 (14.3) |
|  | Carboplatin and liposomal doxorubicin | | 6 (42.9) |  |
|  | Carboplatin, gemcitabine and bevacizumab | | 1 (7.1) |  |
|  | Carboplatin, gemcitabine, and novel experimental agent | | 1 (7.1) |  |
|  | Carboplatin, liposomal doxorubicin and bevacizumab | | 1 (7.1) |  |
|  | Eribulin | |  | 1 (14.3) |
|  | FEC | |  | 1 (14.3) |
|  | Nab paclitaxel | |  | 1 (14.3) |
|  | Paclitaxel and carboplatin | | 3 (21.4) | 1 (14.3) |
|  | Paclitaxel, carboplatin and novel experimental agent | | 1 (7.1) |  |
| Second-line therapy post-disease relapse | | | | |
|  | Carboplatin | |  | 1 (33.3) |
|  | Carboplatin and gemcitabine | | 1 (16.7) |  |
|  | Carboplatin and liposomal doxorubicin | | 1 (16.7) |  |
|  | Carboplatin and vinorelbine | |  | 1 (33.3) |
|  | Cisplatin, liposomal doxorubicin and novel experimental agent | | 1 (16.7) |  |
|  | Epirubicin | |  | 1 (33.3) |
|  | Liposomal doxorubicin | | 1 (16.7) |  |
|  | Novel experimental agent | | 1 (16.7) |  |
|  | Paclitaxel, carboplatin, liposomal doxorubicin and bevacizumab | | 1 (16.7) |  |

AC: doxorubicin/cyclophosphamide

AC-T: doxorubicin/cyclophosphamide/paclitaxel

FEC-D: 5-fluorouracil/epirubicin/cyclophosphamide/docetaxel

FEC-T: fluorouracil/epirubicin/cyclophosphamide/paclitaxel
