# Supplementary material for: Phase 1 trial of olaparib and oral cyclophosphamide in BRCA breast cancer, recurrent BRCA ovarian cancer, non-BRCA triple-negative breast cancer, and non-BRCA ovarian cancer
Source: Br J Cancer. 2019 Jan 17;120(3):279–85. doi: 10.1038/s41416-018-0349-6 (PMC6353881; doi:10.1038/s41416-018-0349-6)
Supplement: Supplementary file 4 — Appendix Tble 3 - Worst grade adverse events overall and by patient cohort at different dose levels [file 41416_2018_349_MOESM4_ESM.docx]

**Appendix Table 3**: Worst grade adverse events overall and by patient cohort at different dose levels

| Worst Grade AE | All patients  N (%) | DL 1  N (%) | | DL 2  N (%) | Extension cohort at DL 2  N (%) | |
| --- | --- | --- | --- | --- | --- | --- |
| 1 | 0 |  |  | | |  |
| 2 | 1 (3.1) |  |  | | | 1 (4.8) |
| 3 | 27 (84.4) | 3 (75.0) | 7 (100) | | | 17 (81.0) |
| 4 | 4 (12.5) | 1 (25.0) |  | | | 3 (14.2) |

AE: adverse event

DL: dose level
