# Supplementary material for: Phase 1 trial of olaparib and oral cyclophosphamide in BRCA breast cancer, recurrent BRCA ovarian cancer, non-BRCA triple-negative breast cancer, and non-BRCA ovarian cancer
Source: Br J Cancer. 2019 Jan 17;120(3):279–85. doi: 10.1038/s41416-018-0349-6 (PMC6353881; doi:10.1038/s41416-018-0349-6)
Supplement: Supplementary file 5 — Appendix Table 4 - Reduction in olaparib dose in different treatment cycle due to Grade 3 or 4 anaemia, neutropenia or thrombocytopenia [file 41416_2018_349_MOESM5_ESM.docx]

**Appendix Table 4**: Reduction in olaparib dose in different treatment cycle due to Grade 3 or 4 anemia, neutropenia or thrombocytopenia

| **Grade 3 and 4 adverse events leading to olaparib dose reduction** | **Total**  **incidence** |  |  |  |  | **Cycle** |  |  |  |
| --- | --- | --- | --- | --- | --- | --- | --- | --- | --- |
|  |  |  | **2** | **3** | **4** | **5** | **6** | **7** | **8** |
| Anemia | 12 | N |  | 1 | 2 | 3 | 4 | 1 | 1 |
|  |  | Dose* |  | 300 to 250 | 500 to 400  600 to 500 | 500 to 250  600 to 300  600 to 500 | 600 to 500  600 to 300 | 500 to 400 | 500 to 250 |
| Neutropenia | 4 | N | 1 | 1 | 2 |  |  |  |  |
|  |  | Dose* | 600 to 500 | 600 to 500 | 600 to 300  500 to 400 |  |  |  |  |
| Thrombocytopenia | 3 | N |  |  | 2 | 1 |  |  |  |
|  |  | Dose* |  |  | 600 to 300  300 to 250 | 500 to 250 |  |  |  |

*Reduction in olaparib dose expressed as total number of milligram per day
