# Supplementary material for: Phase 1 trial of olaparib and oral cyclophosphamide in BRCA breast cancer, recurrent BRCA ovarian cancer, non-BRCA triple-negative breast cancer, and non-BRCA ovarian cancer
Source: Br J Cancer. 2019 Jan 17;120(3):279–85. doi: 10.1038/s41416-018-0349-6 (PMC6353881; doi:10.1038/s41416-018-0349-6)
Supplement: Supplementary file 6 — Appendix Table 5 - Reduction in oral cyclophosphamide dose in different treatment cycles due to Grade 3 or 4 anaemia, neutropenia or thrombocytopenia are described below. In each case, the cyclophospha [file 41416_2018_349_MOESM6_ESM.docx]

**Appendix Table 5**: Reduction in oral cyclophosphamide dose in different treatment cycles due to Grade 3 or 4 anemia, neutropenia or thrombocytopenia are described below. In each case, the cyclophosphamide was reduced from 50mg on Days 1 to 5 to 50mg on Day 1, 3 and 5

|  |  | **Cycle** | | | | | | |
| --- | --- | --- | --- | --- | --- | --- | --- | --- |
| **Grade 3 and 4 adverse events leading to oral cyclophosphamide dose reduction** | **Total**  **incidence** | **2** | **3** | **4** | **5** | **6** | **7** | **8** |
| Anemia | 3 |  | 1 |  | 1 | 1 |  |  |
| Neutropenia | 8 | 3 | 2 | 1 | 1 |  |  | 1 |
| Thrombocytopenia | 4 |  |  | 4 |  |  |  |  |
