# Supplementary material for: Phase 1 trial of olaparib and oral cyclophosphamide in BRCA breast cancer, recurrent BRCA ovarian cancer, non-BRCA triple-negative breast cancer, and non-BRCA ovarian cancer
Source: Br J Cancer. 2019 Jan 17;120(3):279–85. doi: 10.1038/s41416-018-0349-6 (PMC6353881; doi:10.1038/s41416-018-0349-6)
Supplement: Supplementary file 7 — Appendix Table 6 - Relationship with response and platinum sensitivity in the ovarian cancer cohort only [file 41416_2018_349_MOESM7_ESM.docx]

**Appendix Table 6**: Relationship with response and platinum sensitivity in the ovarian cancer cohort only

| Platinum-free interval | RECIST  evaluable  N | RECIST response  N (%) | CA125 evaluable  N | CA125 response  N (%) |
| --- | --- | --- | --- | --- |
| <6 months | 8 | 4 (50.0) | 7 | 3 (42.9%) |
| 6–12 months | 5 | 5 (100.0) | 3 | 2 (66.7%) |
| >12 months | 11 | 9 (81.8) | 10 | 10 (100.0%) |
